# A Longitudinal Ecologic Analysis of Neighborhood-Level Social Inequalities in Health in Texas

**DOI:** 10.3390/ijerph22071076

**Published:** 2025-07-05

**Authors:** Catherine Cubbin, Abena Yirenya-Tawiah, Yeonwoo Kim, Bethany Wood, Natasha Quynh Nhu Bui La Frinere-Sandoval, Shetal Vohra-Gupta

**Affiliations:** 1 Steve Hicks School of Social Work, The University of Texas at Austin, Austin, TX 78712, USA; ayirenya@utexas.edu (A.Y.-T.); natasha.bls@utexas.edu (N.Q.N.B.L.F.-S.); sgupta@austin.utexas.edu (S.V.-G.); 2 Department of Kinesiology, The University of Texas at Arlington, Arlington, TX 76019, USA; yeonwoo.kim@uta.edu; 3 School of Social Work, The University of Texas at Arlington, Arlington, TX 76019, USA; bethany.wood@uta.edu

**Keywords:** neighborhood poverty, social determinants, neighborhood racial composition, heath behaviors, health status

## Abstract

Most health studies use cross-sectional data to examine neighborhood context because of the difficulty of collecting and analyzing longitudinal data; this prevents an examination of historical trends that may influence health outcomes. Using the Neighborhood Change Database, we categorized longitudinal (1990–2010) poverty and White concentration trajectories (long-term low, long-term moderate, long-term high, increasing, or decreasing) for Texas census tracts and linked them to tract-level health-related characteristics (social determinants of health [SDOH] in 2010, health risk and preventive behaviors [HRPB] in 2017, and health status/outcomes [HSO] in 2017) from multiple sources (N = 2961 tracts). We conducted univariate and bivariate descriptive analyses, followed by linear regressions adjusted for population density. SDOH, HRPB, and HSO measures varied widely across census tracts. Both poverty and White concentration trajectories were strongly and consistently associated with a wide range of SDOH. Long-term high-poverty and low-White tracts showed the greatest disadvantages, while long-term low-poverty and high-White tracts had the most advantages. Neighborhoods undergoing changes in poverty or White concentrations, either increasing or decreasing, had less advantageous SDOH compared with long-term low-poverty or long-term high-White neighborhoods. While associations between poverty, White concentration trajectories, and SDOH were consistent, those with HRPB and HSO were less so. Understanding impact of the relationships between longitudinal neighborhood poverty and racial/ethnic composition on health can benefit stakeholders designing policy proposals and intervention strategies.

## 1. Introduction

The relationships between neighborhood-level poverty/racial/ethnic concentrations and health are complex and dynamic. Economically disadvantaged neighborhoods tend to have high concentrations of racial and ethnic minorities, such as those who identify as non-Hispanic Black or Hispanic, and low concentrations of those who identify as non-Hispanic White, largely shaped by decades of institutional racism embedded in housing, lending, and zoning policies and its resulting segregation [1,2,3]. Yet, less is understood about how these neighborhood characteristics change over time and how such changes impact individuals’ well-being, an important gap this study will address. Research of this nature is especially relevant for Texas, one of a handful of majority minority states, meaning that non-Hispanic White residents do not comprise more than 50% of the population [4]. Neighborhood environments are upstream factors that are shaped over time by economic, social, and political forces that may, in turn, determine more downstream determinants of health [5]. These determinants may be conceptualized into social, service, and physical environments [6], with poverty and racial/ethnic concentrations serving as proxy measures. For instance, a neighborhood that has experienced long-term high poverty may differ significantly from one that recently experienced a poverty increase due to disinvestment and social disorganization trends that can affect infrastructure and social cohesion. In contrast, a neighborhood that has experienced long-term low poverty may be different from one that recently experienced a poverty decrease in that it may have greater access to health-promoting goods and services and lesser access to health-damaging goods and services. Cross-sectional measures of neighborhood poverty and racial/ethnic concentrations may fail to capture these nuances.

### 1.1. Neighborhood Poverty and Health

Neighborhood poverty has been found to significantly influence health. While individual-level risk factors such as genetics, family history, socioeconomic status, and behaviors impact health, social determinants of health at the neighborhood level exert strong influence over daily life circumstances that may cumulatively increase one’s chance of becoming ill, physically and mentally [7,8,9,10]. Social determinants of this nature include housing, educational and employment opportunities, transportation, safety, and the availability of nutritional foods and places to be physically active [11]. Poverty at the neighborhood level is thought to be a primary driver of social determinants and can exacerbate structural barriers that contribute to increased health risks [12,13,14,15,16,17,18,19,20,21].

Most research in this area is limited by cross-sectional measures of neighborhood poverty, including recent studies [22,23,24]. Margerison-Zilko et al. [5] and others [25,26] have argued for longitudinal approaches to measuring neighborhood characteristics in health research. These authors postulate that the neighborhood economic context at a given point in time is a result of the long-term interplay of complex social, political, and economic influences and that such historical context can play a role in the health outcomes of its residents. For example, Margerison-Zilko and colleagues [5] found an increased likelihood of preterm birth associated with three different longitudinal measures of neighborhood poverty, but not with a cross-sectional measure of it. Findings from several additional studies with varying health-related outcomes [27,28,29,30] underscore the importance of examining neighborhood poverty trajectories in relation to health. More recent work continues to affirm these patterns found in the prior literature. For example, a study of gentrifying and non-gentrifying tracts across six cities in the U.S. (Chicago, Los Angeles, New York City, Philadelphia, San Francisco, Seattle) found that some neighborhood conditions improved over time in gentrifying neighborhoods, but that the patterns varied based on individual-level race/ethnicity of their residents, whereby Black and Hispanic people experienced worse negative neighborhood conditions on some indicators while Asian and White people experienced better or unchanged conditions [31]. Neighborhood changes have also been linked to weight change. Xiao et al. [32] categorized participants into five different neighborhood “trajectory” groups, finding that living in areas with high poverty concentration was associated with a higher likelihood of substantial weight gain; in contrast, those living in neighborhoods experiencing economic growth were less likely to experience weight gain. Another study examined neighborhood changes in relation to quality-of-life outcomes with data from the Women’s Health Initiative [33]. The researchers classified neighborhoods as “upgrading”, “declining”, or “stable” based on changes in median household income at the census tract level between the 2000 census and the 2007–2011 American Community Survey [33]. The findings indicated that older women living in “upgrading” neighborhoods had significantly better physical functioning-related quality of life compared to those in “stable” or “declining” neighborhoods, with the most pronounced differences observed among those with the lowest household incomes. However, no significant differences were found in self-rated quality of life. These findings demonstrate how neighborhood histories shaped by urban development and disinvestment can influence health.

### 1.2. Neighborhood Racial/Ethnic Composition and Health

Neighborhood racial/ethnic composition also has an impact on health outcomes among various groups, and the relationships are complex. Neighborhood poverty may again play a role, as study findings have indicated that living in poor neighborhoods with high compositions of either Hispanic or Black residents contributes to lower self-rated health, over and above individual-level race/ethnicity [34]. However, Black people living in neighborhoods with high Black compositions reported poor self-rated health across all neighborhood SES levels [34], suggesting how important it is to consider the role of systemic issues like racial discrimination in such contexts. Additionally, studies have found that Black individuals encounter less discrimination in neighborhoods with high compositions of other Black residents compared to neighborhoods with fewer Black residents or more White residents [35,36]. Experiencing this form of discrimination has been found to contribute to depression among Black people [36]. It is also important to consider the historical context of neighborhood racial/ethnic composition as a social determinant of health. A snapshot of the racial/ethnic composition of a neighborhood at any current moment in time may be the product of racially discriminatory housing policies, including urban renewal projects, exclusionary zoning, public housing, and residential redlining [21], accumulated over time. Unlike the aforementioned studies, which examine the history of neighborhood poverty characteristics, there is very little research examining the history of neighborhood racial/ethnic composition in relation to health. In a study of preterm birth among Black and White mothers in Texas [37], the authors found higher odds of preterm birth for mothers living in neighborhoods that experienced long-term high Black or Hispanic concentrations (or long-term low White concentrations) compared to those living in long-term low Black or Hispanic concentrations (or long-term high White concentrations) after controlling for a range of important individual-level confounders.

### 1.3. History of Social Inequality in Texas: A Snapshot

Research examining associations between trajectories of poverty and racial/ethnic composition at the neighborhood level and health may have particular salience for Texas, where deep-rooted patterns of racial segregation and economic inequality have intersected with rapid demographic and economic changes. While segregation and inequality have historically been the norm in Texas, both the racial/ethnic composition and poverty trajectories of different areas have varied significantly over the past several decades, as we discuss below. During the 1990s, immigration patterns led to rapid increases in population growth and marked demographic composition shifts across Texas. Immigrant population growth in major metropolitan areas like Austin (172%) and Dallas (152%) was more than triple the growth in border metropolitan areas such as El Paso (32%) and San Antonio (54%) [38]. Demographic studies indicate that most immigrants living in Texas are Hispanic, while Asians are the fastest-growing racial demographic group in the state [39]. An explanation for the high rates of Hispanic immigrants in Texas can be attributed to the relatively higher birth rates of the Mexican origin population in Texas over time [38,40].

A strong economic outlook in Texas during the 1990s and declining poverty rates overall likely attracted many people, both foreign-born and native-born, to the state [38]. Notable economic disparities in Texas, as in the rest of the U.S., were observed among different racial/ethnic groups, with the 1999 poverty rates for Hispanics, Blacks, and Whites being respectively reported at 25.4%, 23.4%, and 7.8%, along with an approximately $20,000 difference in median household income for Hispanics and Blacks as compared to their more affluent White counterparts. Racial/ethnic differences in wealth, moreover, are more pronounced than income differences [41]. These disparities in economic status among different racial/ethnic groups, along with both economic and racial/ethnic segregation, have implications for health research and neighborhood measures [42,43].

### 1.4. Study Purpose

The purpose of this ecological study is to examine the associations between neighborhood-level longitudinal measures of neighborhood poverty and racial/ethnic concentrations and a broad range of neighborhood-level social determinants of health and health-related characteristics in Texas. Leveraging existing data from multiple sources, particularly the PLACES dataset that provides model-based estimates of health, we investigate how temporally defined community contexts shape health-related outcomes. Specifically, we investigate whether these contexts influence (1) social determinants of health across multiple domains, which subsequently affect (2) health risk and preventive behaviors, and ultimately (3) health status and outcomes following a plausible causal chain [26].

## 2. Materials and Methods

### 2.1. Data

Social Determinants of Health. We utilized the Agency for Healthcare Research and Quality’s social determinants of health (SDOH) dataset from 2010, which includes a wide range of SDOH-related variables. Data sources for these variables are the American Community Survey, Food Access Research Atlas, Centers for Medicare and Medicaid Provider of Services File, and Health Resources and Services Administration’s Medically Underserved Areas. The dataset includes geographically linked variables at the county, ZIP code, and census tract levels for the entire U.S. across five key domains: social context (e.g., age, race/ethnicity), economic context (e.g., income, unemployment rate), education, physical infrastructure (e.g., housing, crime, transportation), and healthcare context (e.g., health insurance). Data from Texas census tracts (N = 5265) for 12 SDOH variables were included for analyses.

Health-related Characteristics. We also utilized CDC data from the 2017 version of the 500 Cities Project, also known as PLACES starting in 2020, which provides modeled health information at the census tract level [44]. The measures presented from this data are derived from multiple data sources, including the Behavioral Risk Factor Surveillance System (BRFSS), the 2010 decennial census, and the American Community Survey (ACS). We categorized the selected measures into health risk and preventive behaviors (HRPB, 7 measures) and health status/outcomes (HSO, 7 measures). Data from Texas census tracts (N = 2961 located in 47 cities) were utilized for analyses. The PLACES project uses a small area estimation (SAE) approach to generate health estimates for adults aged 18 and older at the county, place, census tract, and ZIP Code Tabulation Area (ZCTA) levels. The SAE approach applies a multilevel regression and poststratification method, building a multilevel logistic regression model for each health measure, incorporating sociodemographic variables from the BRFSS, county-level poverty data from the ACS, and state- and county-level random effects [44]. The model is then used to calculate the predicted probability of each outcome for each geographic level. To enhance accuracy, Monte Carlo simulations were used to generate point estimates and 95% confidence intervals [44].

**Figure 1:**
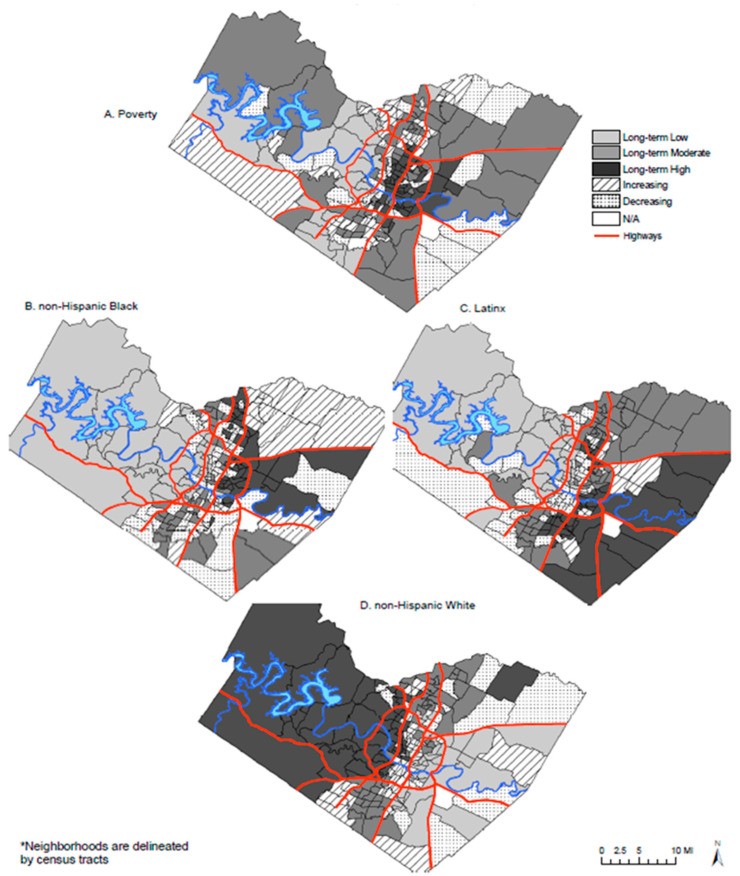
Neighborhood-level * trajectories categorized by poverty and racial/ethnic concentrations, Travis County, TX (1990–2010).

Trajectories. We obtained data on the economic (% poor, as a measure of socioeconomic disadvantage) and racial/ethnic (% White, as a measure of social advantage) compositions of all Texas census tracts for the years 1990, 2000, and 2010 from the Neighborhood Change Database (NCDB) [45]. The NCDB provides census tract-level data from the U.S. decennial censuses and the American Community Survey. To account for changes in census tract boundaries over time, census tract data in the NCDB were recalculated and weighted so all years of data correspond to the geographic census tract boundaries in 2000. This allowed for comparisons of the same geographic boundaries over time. We did not include data from 1980 or 1970 because nearly a quarter (1277 out of 5265) of census tracts in Texas had missing data for at least one of the variables of interest in each of the years or both. (Note: The U.S. was not completely divided into census tracts until 1990, so the missing data in TX are primarily in rural areas.) Of the total 5265 Texas census tracts, we excluded 69 tracts missing poverty data in 1990, 2000, or 2010 and 29 tracts with no discernable trajectory pattern across the trajectory variables (operationalization of measures are detailed below), resulting in 5167 tracts for analyses. For validation and visual purposes, we mapped the poverty and racial/ethnic trajectories in Travis County, TX, an area with which we are familiar (Figure 1).

For the final analytic sample of tracts, we merged the three datasets, resulting in our final analytic observation of 2961 census tracts (all located in urbanized areas).

### 2.2. Measures

The 12 SDOH measures included distance to nearest emergency department and obstetrics department; housing characteristics, including value, renter occupancy, and crowding; socioeconomic measures including income inequality, less than high school and Bachelor’s education, public assistance, and unemployment; and demographic measures, including limited English proficiency and single-parent households. Health risk and preventive behaviors (HRPB) measures included sleep, smoking, physical activity, hypertension, dental and routine check-ups, and insurance status. Health status/outcome (HSO) measures included cancer, pulmonary disease, heart disease, diabetes, obesity, and poor mental or physical health status. Finally, neighborhood poverty and White concentration trajectories were defined as follows: cross-sectional measures of each, based on the distribution of the 1990, 2000, and 2010 census tract data (0–5%, 5–20%, or >20% for poverty, tertiles for White), were examined and categorized into five trajectories defined a priori [5]: (1) long-term low, (2) long-term moderate, (3) long-term high, (4) increasing, and (5) decreasing. The Supplementary Materials summarizes the measures with definitions, data source, and year of data collection.

### 2.3. Analytical Plan

First, we examined univariate statistics for all variables and then conducted bivariate analysis of variance (i.e., ANOVA) between the poverty/White trajectories and each dependent variable (SDOH, HRPB, HSO). Next, we estimated associations between the poverty or White trajectory and SDOH/HRPB/HSO measures using linear regression models, controlling for population density (quartiles) obtained from the 2010 Neighborhood Change Database. Distance to nearest emergency department and obstetrics department, housing value, crowded housing, public assistance, and limited English proficiency were first log-transformed to account for the high degree of skewness. Because of multiple testing, we used the more restrictive *p* value of 0.001 to infer statistical significance through a Bonferroni correction. We used SAS software version 9.4 (SAS Institute, Cary, NC, USA) for all analyses.

## 3. Results

Table 1 presents medians and ranges (for the continuous dependent variables) or percent distributions (for the trajectories) for the 2961 Texas census tracts. SDOH varied widely across the tracts. Half of the residents lived over five miles from the closest emergency department (range 0–52 miles) and over three miles from the closest obstetrics department (range 0–46 miles). Regarding housing characteristics, the median home value was $114,000, half of the tracts had more than a third of the units occupied by renters, and crowded housing ranged from 0% to 40%. The median income inequality value was 0.4; half of the tracts had over 18% of residents without a high school degree, and half of the tracts had over 14% of residents with a Bachelor’s degree. Tracts had from 0–65% of residents with some sort of public assistance, the median unemployment value was 6.5%, and 0–100% of tracts had households with limited English proficiency. Finally, the median value of single-parent households was nearly 30%.

The lowest median value among the HRPB measures was 17% for smoking, and the highest median value was 67% for having had a routine checkup in the past year. Having had a dental visit was not far behind at 57%, while the other measures had similar median values (26–35%). Some tracts had very high and concerning values; for instance, the highest level of hypertension or uninsurance rate in a tract was nearly two-thirds. Among the HSO measures, the lowest median values were for cancer, pulmonary disease, and heart disease (about 5%), diabetes and poor mental or physical health status median values were about twofold higher (11–12%), and obesity had the highest median value by far at 33%. As with the HRPB measures, some tracts had very high and concerning values for the HSO measures; for instance, the highest level of obesity was over 50%, the highest level of diabetes was nearly 30%, and the highest level of cancer and heart disease was over 20%, indicating a high chronic disease burden in some communities.

During the 1990–2010 time period, about a quarter of the tracts experienced long-term high poverty, 30% experienced long-term moderate poverty, and 11% experienced long-term low poverty, while 21% and 14% experienced an increase or decrease in poverty, respectively. In terms of White trajectories, about one-fifth experienced either long-term high or moderate White concentrations, and nearly one-third experienced long-term low White concentrations, while 13% and 16% experienced an increase or decrease in their White population, respectively.

Bivariate analyses using ANOVA demonstrated statistically significant relationships between both the poverty and White trajectories and every SDOH measure (see Supplementary Materials). In contrast, while all but one health-related measure (smoking) was significantly associated with the poverty trajectories, only two health-related measures (smoking, obesity) were statistically associated with the White trajectories. In contrast, most of the health-related measures were associated with the Black trajectories, and about half of the measures were associated with the Hispanic trajectories (see Supplementary Materials).

Table 2 presents the results from the linear regression models that examined associations between the poverty trajectories and each dependent variable. Starting with the SDOH, compared with long-term low poverty tracts and adjusting for population density, all other poverty trajectory categories were further away from the nearest emergency department, but there were no statistically significant distances from the nearest obstetrics department. In terms of housing characteristics, median home values were lower and renter-occupied or crowded housing units were higher in all poverty trajectory categories compared with long-term low poverty tracts. For example, long-term high poverty tracts had 27% more renter-occupied housing units compared with long-term low poverty tracts after adjusting for population density. In terms of socioeconomic characteristics, every poverty trajectory category was significantly higher (income inequality, less than high school, public assistance, unemployed) or lower (Bachelor’s degree) compared with long-term low poverty tracts (after adjusting for population density). Similarly, all poverty trajectory categories had higher levels of limited English proficiency and single-parent households compared to long-term low poverty tracts. Interestingly, both increasing and decreasing poverty trajectories had beta coefficients in the same direction, indicating that changes in neighborhood poverty, whether increasing or decreasing, were associated with more disadvantaged SDOH compared with long-term low poverty neighborhoods.

Compared with SDOH measures, fewer of the HRPB or HSO measures (and/or fewer poverty trajectory categories) were significantly associated with the poverty trajectories after adjusting for population density. Among the HRPB measures, long-term moderate (no physical activity, hypertension), long-term high (hypertension, routine checkup), or increasing (no physical activity, hypertension) poverty trajectories had positive associations with the HRPB measures after adjusting for population density. For instance, each poverty trajectory category was associated with about a 2% increase in hypertension compared with long-term low poverty tracts after adjusting for population density. With the exception of poor mental health status, all HSO measures were associated with at least one of the poverty trajectory categories. All poverty trajectory categories were associated with higher rates of coronary heart disease compared with long-term low poverty tracts, while all but the decreasing poverty tracts were associated with more chronic obstructive pulmonary disease and diabetes. Only the long-term high poverty tracts were associated with increased cancer rates, while only long-term moderate poverty tracts were associated with higher obesity. Additionally, both long-term moderate and increasing poverty tracts were associated with higher levels of poor physical health status after adjusting for population density.

Table 3 presents the results from the linear regression models that examined associations between the White trajectories and each dependent variable. Whereas distance to the nearest emergency or obstetrics department was largely unrelated to the White trajectories, each housing measure was related to the trajectories, with each category associated with lower home values or higher proportions of renter-occupied and crowded housing compared with long-term high White tracts. For example, long-term low White tracts had about 1.6% more crowded housing compared with long-term high White tracts after adjusting for population density. Increasing White tracts had higher income inequality, and decreasing White tracts had lower income inequality compared with long-term high White tracts. Every other SDOH measure was associated with every White trajectory category. For example, long-term low White tracts had 28% more people with less than a high school degree, 17% fewer people with a Bachelor’s degree, 5% more unemployed people, and 16% more single-parent households compared with long-term high White tracts, after adjusting for population density.

In contrast to the poverty trajectories, very few of the HRPB or HSO measures were statistically significant with the White trajectories, and only for the decreasing White trajectories. These tracts, compared with long-term high White tracts, were associated with higher levels of insufficient sleep, no physical activity, uninsured people, and diabetes, after adjusting for population density.

For both the poverty and White trajectory measures, population density was significantly associated with nearly every measure, demonstrating the strong effect that urbanicity has on SDOH and health-related measures. The Black and Hispanic trajectory models are included in the Supplementary Materials. In brief, nearly all the SDOH were associated with the Black and Hispanic trajectories. The long-term high Black trajectory category (compared with long-term low) was associated with 8 out of the 14 health-related measures, while the Hispanic trajectory categories were by and large not associated with the health-related measures. As with the poverty and White trajectories, population density was associated with nearly every measure.

## 4. Conclusions

We found that both poverty and White concentration trajectories were strongly and consistently associated with a wide range of SDOH, including health care availability, housing characteristics, socioeconomic characteristics, and household characteristics. Neighborhoods characterized as long-term high poverty consistently experienced the greatest disadvantages compared to those characterized as long-term low poverty, with long-term moderate poverty neighborhoods falling in between. A similar pattern was observed for neighborhoods characterized as long-term low or moderate White compared with long-term high White; neighborhoods that have high concentrations of White people historically had the most advantageous SDOH. An interesting finding is that neighborhoods undergoing changes in poverty or White concentrations, whether increasing or decreasing, had less favorable SDOH compared with long-term low poverty or long-term high White neighborhoods. In most cases, differences from long-term low poverty (or high White) neighborhoods were typically larger for areas with rising poverty (or declining White populations) than for those with moderate long-term poverty (or White concentrations). A prior study of neighborhood change in relation to SDOH is consistent with our findings [31].

While the associations between poverty and White concentration trajectories and SDOH measures were quite consistent, the associations with measures of HRPB and HSO were less so. Although the poverty trajectories were associated with most of the HSO measures and a few of the HRPB measures, fewer of the poverty trajectory categories were significant. For example, only the long-term high poverty category of neighborhoods was significantly associated with higher rates of cancer. For the most part, White trajectories were mostly unrelated to the HRPB and HSO measures, with the exception of decreasing White neighborhoods (vs. long-term high White neighborhoods) having a significant association with higher rates of four measures (insufficient sleep, no physical activity, uninsured, diabetes). In supplementary analyses with Black or Hispanic trajectories (see Supplementary Materials), we found that most of the HRPB and HSO measures were associated with long-term high Black trajectory neighborhoods (compared with long-term low Black neighborhoods), while there were basically no statistically significant associations with any of the Hispanic trajectory categories. This finding suggests that, unlike poverty trajectories, racial/ethnic concentrations may have a limited or an indirect influence on population-level health indicators, and the associations depend on which racial/ethnic group is being considered. A possible explanation for the divergence in findings is that the racial/ethnic trajectories were much more variable than the poverty trajectories in terms of the health-promoting or damaging factors within each category. In other words, for instance, some long-term high Hispanic neighborhoods may be protective and other may be risky for health, therefore resulting in a null finding. An alternative approach we could have considered would have been to examine whether the SDOH were associated with the HRPB and HSO measures rather than the poverty and racial/ethnic trajectories predicting the health-related measures.

Our literature review identified two studies that used the PLACES dataset to examine neighborhood-level differences in SDOH and health characteristics and share some similarities with ours; Refs. [24,46] Li, Douglas, and Subica (2023) [47] examined majority Asian, Black, Hispanic, and White tracts in southern California and found that “minority” neighborhoods had more SDOH and health disadvantages compared with White neighborhoods and that minority neighborhoods were associated with higher rates of fair/poor health status after adjusting for several demographic and socioeconomic characteristics. Additionally, Liu and colleagues [24] examined neighborhood-level cardiovascular risk factors and disease in relation to environmental burden among all U.S. census tracts and found that associations between environmental burden and cardiovascular health was strongest in neighborhoods that experienced the most social vulnerability [24].

Our findings revealed that approximately a fourth of neighborhoods in Texas experienced high poverty from 1990 to 2010, and about a fifth of neighborhoods experienced increases in poverty over the 20-year timespan. Additionally, roughly 40% of neighborhoods experienced concentrations of the White population (long-term moderate or high), and another 13% were increasing in their concentration of the White population. When considering the intersection of the poverty and White trajectories, they tended to co-occur. For example, 62% of long-term low poverty tracts in Texas are also long-term high White tracts, and 71% of long-term high poverty tracts are also long-term low White tracts (see see Supplementary Materials). We conducted an ancillary logistic regression analysis and found that long-term low White neighborhoods were significantly associated with greater odds of having a negative poverty trajectory (OR: 17.75, 95% CI: 14.67–21.48), defined as either long-term high or increasing in poverty, compared to long-term high White neighborhoods. Moreover, neighborhoods with a decreasing White population were also significantly associated with increased odds of having a negative poverty trajectory (OR: 4.90, 95% CI: 3.98–6.03). These entrenched patterns, which are replicable and have important implications for health and other social outcomes, would be impossible to observe without a longitudinal analysis such as the one we conducted.

The incidence of neighborhoods experiencing persistently high and increasing poverty rates is concerning given that long-term neighborhood poverty characteristics are related to health (and other) outcomes. For example, women living in long-term high poverty neighborhoods had higher odds of obesity than women living in long-term low poverty neighborhoods [48]. These results are congruent with a research study using a latent class analysis to categorize neighborhood poverty trajectories, which revealed that children from historically high poverty neighborhoods were more likely to experience lower quality of sleep than their peers from historically moderate or low poverty neighborhoods [30]. Additional studies have shown that negative poverty characteristics of neighborhoods are related to poor outcomes of smoking abstinence and attitudes towards cessation [48], sexually transmitted infections and intentional injuries [49], changes in body weight [50,51], hypertension [52,53], kidney disease [54], asthma [55], and general health or wellbeing [28,56,57,58]. Importantly, these studies showed relationships between neighborhood poverty and health outcomes after accounting for individual-level indicators of socioeconomic status (SES), including income level, occupation, and/or education.

The relatively constant White compositions of neighborhoods over the 20–year timespan (over 70% of tracts were in the long-term low, moderate, or high White categories) could suggest a general lack of neighborhood diversity in Texas. A recent study by members of our team supports this claim, in that our cluster analysis of all tracts in Texas found that only 3% of all tracts in Texas (N = 167) are diverse in terms of multiple races/ethnicities [59]. High concentrations of single racial/ethnic populations in neighborhoods may indicate signs of residential segregation at the metropolitan scale, as observed in the maps of major Texas cities [60]. These residential patterns should be noted, given the associations between segregation and health outcomes and, more importantly, how they differ by race/ethnicity. For example, adverse birth outcomes among Black mothers were associated with living in segregated Black neighborhoods, whereas no association was found between segregated White neighborhoods and adverse birth outcomes for White mothers [61]. Differentiation of the impact of segregation on health by individual’s race was also found in a study examining cardiovascular health, with higher risk of adverse health outcomes being found for Black participants living in segregated Black neighborhoods when contrasted with their Black peers living in less segregated neighborhoods [62]. In contrast, they revealed health-promoting associations between being White and living in a segregated White neighborhood, while no connection was found between segregation in Hispanic neighborhoods and cardiovascular health risks for Hispanic participants.

The concentration of racial/ethnic minorities over time, particularly Black and Hispanic populations, in disadvantaged communities is a well-studied phenomenon that is largely the byproduct of inequitable housing policies and discriminatory practices by economic institutions [63]. These neighborhoods are particularly susceptible to economic disinvestment, which has led to declines in infrastructure, housing conditions, educational quality, and quality of life [64]. Research suggests that the disproportionate share of Black and Hispanic populations living in high poverty neighborhoods contributes to racial/ethnic health disparities [65]. For instance, such neighborhoods are more likely to contain food deserts [66], lack access to supermarkets [67,68], and have a shortage of physical activity facilities [69]. However, a majority of the neighborhood/health research lacks a longitudinal approach, and a growing body of work, including the current study, suggests that historical context matters.

Our study is not without limitations. We used a very conservative *p*-value (0.001) to infer statistical significance, given the high number of tests we ran. Using a more conventional threshold of 0.05, we would have made many more conclusions of statistical significance for the health-related measure associations. Our trajectories measures were based on a priori considerations, and alternative measures of neighborhood histories may have yielded different results. However, we visually inspected maps of Travis County, a place that is familiar to us, as a means of validating the measures. Because Texas overall is a very diverse state and for the sake of brevity, we used only one indicator of racial/ethnic concentration (White concentration) rather than other measures such as Black and/or Hispanic concentration, which are more commonly used in the literature to indicate “minority” concentration or social disadvantage. While a low concentration of the White population does not specify which racial/ethnic groups are present, we found it impractical to include multiple racial/ethnic concentration measures, given the number of dependent variables under investigation. We also intentionally included White concentration to indicate social advantage along with poverty concentration as an indicator of social disadvantage. The BRFSS measures are self-reported and subject to recall and social desirability biases, and because our study is ecological, inferences at the individual level cannot be made (ecological fallacy). The primary limitations of the small area estimation approach for our purposes are that population estimates at the tract level used to make estimates are based on 2010 decennial census data, but the outcomes are from the 2017 PLACES dataset; in addition, poverty and race are used as some of the variables in calculating estimates, potentially adding collinearity to our models. Finally, our study was based on data from urbanized areas of Texas and, therefore, may not be generalizable to other states or less urbanized areas. It would be difficult to replicate this study in the most rural areas because the PLACES data is only available for tracts with at least 50 adults (current data release) in order to produce reliable estimates; our findings may be generalizable to other large, diverse states though, such as California and Florida, but replication of our methodology would be required. Nevertheless, our findings demonstrate that neighborhood poverty and White trajectories have salient implications for SDOH and health. Additionally, this study bolsters other notable strengths by examining 20 years of neighborhood data (1990–2010) to construct poverty and racial/ethnic concentration trajectories rather than relying on cross-sectional data. By linking these longitudinal trajectories to a broad range of health-related outcomes, our study offers one of the most comprehensive ecological assessments of neighborhood change and health to date in Texas. Our study design may serve as a useful model for similar investigations into neighborhood and health equity in other states. Furthermore, the results of this study not only advance the literature but can inform targeted place-based interventions across the state by accounting for both the historical context and the state’s evolving health equity landscape.

Our findings point to the need for place-based policies that address the structural and environmental conditions shaping health. Recent efforts across Texas, such the Dallas Racial Equity plan or Austin’s equity-based budgeting, illustrate a growing policy recognition of the need to address the long-term effects of racial and economic segregation on neighborhood health. These initiatives support the argument that understanding historical trajectories of poverty and racial/ethnic composition is essential for designing equitable and effective public health strategies. Policy recommendations for long-term high or increasing poverty include funding federally qualified health centers (FQHCs) and mobile health clinics. While these interventions exist in some regions of Texas, a broader commitment through federal/state funding as well as private funding is needed to expand access. Private/public partnerships for policy development and implementation would work well in states like Texas. For example, H-E-B public health grants, along with WIC/SNAP Double Up programs supported by the Sustainable Food Center and the Texas Hunger Initiative, help improve both nutrition and economic stability for working families. Designating neighborhoods with long-term poverty or increasing poverty as health investment zones can serve as a mechanism to direct resources to health infrastructure such as subsidized fitness facilities, culturally tailored nutrition programs, community walking clubs, and increased green spaces and sidewalks. These investments foster community safety, improve family well-being, boost workforce productivity, and strengthen local infrastructure in historically underserved areas. At the same time, attention must be paid to preventing the unintentional consequences of gentrification that may result from such investment, such as displacement of long-term or marginalized residents.

An in-depth understanding of the relationships between longitudinal neighborhood poverty and White composition trajectories and health can be of great benefit to stakeholders tasked with designing policy proposals and intervention strategies. Public health campaigns can be designed to address specific issues common to those living in neighborhoods with long-term poverty trajectories and/or particular racial/ethnic compositions. For example, it is feasible to consider that food deserts may have the strongest adverse impact upon individuals living in neighborhoods with long-term poverty trajectories. If so, this may help explain Sheehan et al.’s (2017) [48] findings, which found higher odds of obesity among women living in long-term high poverty neighborhoods than in their counterparts from long-term low poverty neighborhoods. Barriers of this nature could be addressed via collaborations between those in the fields of community infrastructure and planning, business, food systems, and transportation.

Rather than a one-size-fits-all approach to public health campaigns targeting obesity, different versions could be customized to account for socioeconomic status, neighborhood access, and the cultural preferences of specific racial/ethnic groups, such as by proposing more nutritionally beneficial recipes of traditional dishes made from affordable and locally accessible ingredients. These tailored public health nutrition programs are supported by our finding that long-term high-poverty neighborhoods are associated with higher obesity and lower education attainment.

Taken together, these insights emphasize that neighborhood-level poverty and racial segregation have lasting health consequences that are not easily reversed. Policies and interventions must therefore account for the long-term trajectories of place-based disadvantage. We encourage further research that examines the intersection of poverty and racial/ethnic composition from a longitudinal perspective, which can help guide the creation and implementation of targeted strategies to address the specific needs of communities with distinct historical patterns of structural disadvantage.

## Figures and Tables

**Table 1:** Prevalence of social determinants of health, health-related characteristics, and trajectories, N = 2961 census tracts, Texas.

	Median (Range) or % of Total
**Social Determinants of Health**	
Distance to the nearest emergency department (miles)	4.6 (0.1–51.6)
Distance to the nearest obstetrics department (miles)	3.2 (0.0–45.6)
Median home value ($)	113,500 (9999–1,000,001)
Rented occupied housing units (%)	32.5 (0.0–100)
Crowded housing (%)	3.6 (0.0–39.8)
Gini index of income inequality	0.4 (0.0–0.7)
Less than high school (%)	17.8 (0.0–100)
Bachelor’s degree (%)	13.8 (0.0–61.4)
Public assistance income or food stamps/SNAP (%)	8.5 (0.0–64.8)
Unemployed (%)	6.5 (0.0–59.7)
Limited English proficiency (%)	5.8 (0.0–100)
Single-parent households (%)	29.4 (0.0–100.0)
**Health Risk and Preventive Behaviors**	
Insufficient sleep (%)	34.8 (21.7–49.0)
Current smoking (%)	16.6 (4.5–40.6)
No physical activity (%)	30.4 (13.6–52.2)
Hypertension (%)	29.7 (7.7–62.9)
Dental Visit (%)	57.0 (23.9–85.5)
Routine checkup (%)	66.5 (50.8–86.9)
Uninsured (%)	25.5 (5.8–64.1)
**Health Status/Outcomes**	
Cancer (%)	4.8 (0.7–20.9)
Chronic obstructive pulmonary disease (%)	5.5 (1.3–18.2)
Coronary heart disease (%)	5.4 (0.4–20.9)
Diabetes (%)	10.6 (1.2–29.4)
Obesity (%)	33.1 (15.8–52.3)
Poor mental health status (%)	11.7 (4.8–21.8)
Poor physical health status (%)	12.1 (4.0–30.2)
**Poverty trajectories (%)**	
Long-term low	11.4
Long-term moderate	30.1
Long-term high	24.2
Increasing	20.7
Decreasing	13.7
**White trajectories (%)**	
Long-term low	32.0
Long-term moderate	20.2
Long-term high	19.0
Increasing	12.8
Decreasing	16.1

**Table 2:** Linear regression models examining associations between poverty trajectories and social determinants of health and health-related characteristics, N = 2961 census tracts, Texas.

	Long-Term Moderate ^a^	Long-Term High ^a^	Increasing ^a^	Decreasing ^a^	Population Density (Quartile 1) ^b^	Population Density (Quartile 2) ^b^	Population Density (Quartile 3) ^b^	R^2^	F (*p* Value)
	beta (se)	beta (se)	beta (se)	beta (se)	beta (se)	beta (se)	beta (se)		
Social Determinants of Health									
Distance to the nearest emergency department ^c^	0.423 (0.053) *	0.317 (0.054) *	0.282 (0.056) *	0.351 (0.061) *	1.069 (0.045) *	0.463 (0.044) *	0.132 (0.044)	0.218	118.70 (<0.0001)
Distance to the nearest obstetrics department ^c^	−0.002 (0.049)	−0.144 (0.051)	−0.006 (0.052)	0.005 (0.058)	1.054 (0.042) *	0.209 (0.041) *	0.030 (0.041)	0.234	129.82 (<0.0001)
Median home value ^c^	−0.489 (0.038) *	−1.010 (0.038) *	−0.555 (0.039) *	−0.524 (0.043) *	−0.190 (0.025) *	−0.066 (0.024)	−0.007 (0.024)	0.313	150.99 (<0.0001)
Rented occupied housing units	15.197 (1.791) *	27.306 (1.798) *	20.309 (1.840) *	12.679 (2.013) *	−24.102 (1.176) *	−11.294 (1.140) *	−8.969 (1.122) *	0.269	122.53 (<0.0001)
Crowded housing ^c^	1.039 (0.082) *	1.852 (0.082) *	1.229 (0.084) *	0.992 (0.092) *	−0.462 (0.054) *	−0.353 (0.052) *	−0.333 (0.051) *	0.254	113.30 (<0.0001)
Gini index of income inequality	0.032 (0.006) *	0.077 (0.006) *	0.041 (0.006) *	0.032 (0.006) *	0.012 (0.004) *	−0.003 (0.004)	−0.003 (0.003)	0.124	47.50 (<0.0001)
Less than high school	12.390 (1.149) *	33.433 (1.153) *	15.462 (1.180) *	15.045 (1.291) *	−5.220 (0.754) *	−4.599 (0.731) *	−4.538 (0.720) *	0.408	228.48 (<0.0001)
Bachelor’s degree	−15.599 (0.799) *	−24.425 (0.802) *	−15.829 (0.821) *	−14.453 (0.898) *	−2.777 (0.525) *	−0.237 (0.509)	0.863 (0.501)	0.331	163.80 (<0.0001)
Public assistance income or food stamps/SNAP	1.275 (0.069) *	2.326 (0.069) *	1.453 (0.071) *	1.196 (0.078) *	−0.036 (0.045)	−0.083 (0.044)	−0.131 (0.043)	0.394	215.30 (<0.0001)
Unemployed	2.195 (0.365) *	5.610 (0.366) *	3.295 (0.375) *	2.130 (0.410) *	−1.061 (0.240) *	0.234 (0.232)	0.141 (0.229)	0.162	64.72 (<0.0001)
Limited English proficiency ^c^	0.698 (0.086) *	1.621 (0.086) *	0.997 (0.088) *	0.741 (0.096) *	−1.135 (0.056) *	−0.605 (0.055) *	−0.466 (0.054) *	0.321	156.48 (<0.0001)
Single-parent households	14.176 (1.396) *	26.667 (1.400) *	17.639 (1.434) *	10.779 (1.569) *	−8.667 (0.917) *	−1.072 (0.888)	−1.644 (0.875)	0.224	95.96 (<0.0001)
Health Risk and Preventive Behaviors									
Insufficient sleep	0.752 (0.287)	0.743 (0.296)	0.617 (0.305)	0.234 (0.337)	1.982 (0.247) *	1.601 (0.240) *	0.930 (0.241) *	0.026	12.14 (<0.0001)
Current smoking	0.469 (0.311)	0.378 (0.321)	0.446 (0.330)	0.210 (0.366)	1.584 (0.268) *	1.173 (0.261) *	0.409 (0.261)	0.014	6.78 (<0.0001)
No physical activity	2.080 (0.526) *	1.631 (0.543)	1.916 (0.559) *	1.695 (0.618)	2.934 (0.454) *	3.141 (0.441) *	1.798 (0.441) *	0.024	11.28 (<0.0001)
Hypertension	2.039 (0.432) *	2.062 (0.446) *	1.941 (0.459) *	1.101 (0.507)	2.911 (0.372) *	2.290 (0.362) *	1.121 (0.362)	0.030	14.24 (<0.0001)
Dental visit	−2.232 (0.863)	−0.878 (0.891)	−2.308 (0.917)	−1.387 (1.013)	−2.967 (0.744) *	−3.409 (0.723) *	−1.710 (0.723)	0.010	5.18 (<0.0001)
Routine checkup	0.500 (0.251)	0.957 (0.259) *	0.276 (0.266)	0.118 (0.295)	1.013 (0.217) *	0.253 (0.210)	0.173 (0.211)	0.013	6.54 (<0.0001)
Uninsured	2.575 (0.841)	0.974 (0.868)	2.731 (0.893)	2.536 (0.988)	2.460 (0.725) *	3.659 (0.705) *	2.276 (0.705)	0.012	6.17 (<0.0001)
Health Status/Outcomes									
Cancer	0.272 (0.103)	0.354 (0.106) *	0.245 (0.109)	0.167 (0.121)	0.245 (0.089)	0.084 (0.086)	−0.027 (0.086)	0.006	3.49 (0.001)
Chronic obstructive pulmonary disease	0.551 (0.125) *	0.429 (0.129) *	0.477 (0.133) *	0.325 (0.147)	0.695 (0.108) *	0.613 (0.105) *	0.280 (0.105)	0.023	10.79 (<0.0001)
Coronary heart disease	0.757 (0.131) *	0.619 (0.135) *	0.671 (0.139) *	0.516 (0.153) *	0.637 (0.113) *	0.696 (0.109) *	0.360 (0.110) *	0.025	11.85 (<0.0001)
Diabetes	1.433 (0.280) *	1.280 (0.290) *	1.353 (0.298) *	0.924 (0.329)	1.234 (0.242) *	1.515 (0.235) *	0.868 (0.235) *	0.021	10.24 (<0.0001)
Obesity	1.440 (0.407) *	1.218 (0.420)	1.346 (0.43)	1.227 (0.478)	2.955 (0.351) *	2.646 (0.341) *	1.440 (0.341) *	0.033	15.23 (<0.0001)
Poor mental health status	0.481 (0.191)	0.169 (0.197)	0.452 (0.203)	0.379 (0.224)	0.657 (0.165) *	0.732 (0.160) *	0.446 (0.160)	0.009	5.00 (<0.0001)
Poor physical health status	1.325 (0.282) *	0.868 (0.291)	1.202 (0.300) *	1.015 (0.331)	1.274 (0.243) *	1.553 (0.237) *	0.883 (0.237) *	0.021	10.15 (<0.0001)

* *p* ≤ 0.001 based on a Bonferroni correction for multiple tests. ^a^ Reference group is long-term low; ^b^ Reference group is Quartile 4 (highest); ^c^ log-transformed.

**Table 3:** Linear regression models examining associations between White trajectories and social determinants of health and health-related characteristics, N = 2961 census tracts, Texas.

	Long-Term Low ^a^	Long-Term Moderate ^a^	Increasing ^a^	Decreasing ^a^	Population Density (Quartile 1) ^b^	Population Density (Quartile 2) ^b^	Population Density (Quartile 3) ^b^	R^2^	F (*p* Value)
	beta (se)	beta (se)	beta (se)	beta (se)	beta (se)	beta (se)	beta (se)		
Social Determinants of Health									
Distance to the nearest emergency department ^c^	−0.073 (0.062)	−0.110 (0.064)	−0.016 (0.073)	−0.151 (0.071)	1.035 (0.054) *	0.473 (0.049) *	0.138 (0.049)	0.194	80.36 (<0.0001)
Distance to the nearest obstetrics department ^c^	−0.053 (0.059)	−0.189 (0.061)	−0.244 (0.070) *	−0.089 (0.067)	1.090 (0.051) *	0.217 (0.047) *	0.044 (0.046)	0.229	98.82 (<0.0001)
Median home value ^c^	−0.772 (0.031) *	−0.297 (0.032) *	−0.191 (0.032) *	−0.348 (0.035) *	−0.415 (0.027) *	−0.097 (0.025) *	−0.031 (0.024)	0.291	136.33 (<0.0001)
Rented occupied housing units	10.584 (1.522) *	7.967 (1.572) *	10.584 (1.781) *	7.571 (1.722) *	−23.492 (1.316) *	−12.406 (1.206) *	−9.626 (1.186) *	0.190	78.28 (<0.0001)
Crowded housing ^c^	1.585 (0.064) *	0.631 (0.066) *	0.506 (0.075) *	0.855 (0.072) *	0.001 (0.055)	−0.281 (0.051) *	−0.276 (0.050) *	0.298	140.62 (<0.0001)
Gini index of income inequality	0.006 (0.005)	−0.006 (0.005)	0.021 (0.005) *	−0.024 (0.005) *	0.003 (0.004)	−0.009 (0.004)	−0.005 (0.004)	0.0455	16.42 (<0.0001)
Less than high school	27.690 (0.916) *	6.355 (0.947) *	5.722 (1.073) *	11.855 (1.037) *	3.076 (0.793) *	−2.989 (0.726) *	−3.286 (0.714) *	0.422	241.19 (<0.0001)
Bachelor’s degree	−16.850 (0.644) *	−5.614 (0.665) *	−3.087 (0.754) *	−7.127 (0.729) *	−8.099 (0.557) *	−0.853 (0.510)	0.301 (0.502)	0.333	165.38 (<.0001)
Public assistance income or food stamps/SNAP	1.644 (0.058) *	0.582 (0.060) *	0.473 (0.067) *	0.676 (0.065) *	0.406 (0.050) *	−0.035 (0.456)	−0.085 (0.045)	0.351	178.89 (<0.0001)
Unemployed	4.625 (0.295)*	1.319 (0.305) *	1.443 (0.346) *	1.950 (0.334) *	0.037 (0.255)	0.415 (0.234)	0.295 (0.230)	0.157	62.58 (<0.0001)
Limited English proficiency ^c^	1.940 (0.063) *	0.899 (0.065) *	0.715 (0.073) *	1.351 (0.071) *	−0.543 (0.054) *	−0.479 (0.050) *	−0.379 (0.049) *	0.443	263.14 (<0.0001)
Single-parent households	16.492 (1.183) *	7.433 (1.222) *	8.513 (1.384) *	7.175 (1.339) *	−5.677 (1.023) *	−1.143 (0.937)	−1.541 (0.922)	0.144	56.45 (<0.0001)
Health Risk and Preventive Behaviors									
Insufficient sleep	0.564 (0.261)	0.459 (0.274)	0.209 (0.307)	1.242 (0.300) *	2.263 (0.258) *	1.626 (0.241) *	0.951 (0.241) *	0.029	13.40 (<0.0001)
Current smoking	0.093 (0.283)	−0.219 (0.298)	0.260 (0.333)	0.821 (0.325)	1.702 (0.280) *	1.184 (0.262) *	0.430 (0.262)	0.017	8.17 (<0.0001)
No physical activity	1.080 (0.479)	0.798 (0.504)	1.648 (0.464)	1.834 (0.550) *	3.393 (0.474) *	3.143 (0.444) *	1.822 (0.444) *	0.023	10.89 (<0.0001)
Hypertension	0.653 (0.394)	0.209 (0.415)	0.566 (0.464)	1.078 (0.453)	3.108 (0.390) *	2.179 (0.365) *	1.056 (0.365)	0.023	10.92 (<0.0001)
Dental visit	−0.455 (0.785)	−0.567 (0.826)	−1.412 (0.924)	−2.519 (0.902)	−3.329 (0.777) *	−3.367 (0.727) *	−1.701 (0.727)	0.010	5.20 (<0.0001)
Routine checkup	0.365 (0.229)	0.190 (0.241)	−0.097 (0.270)	0.191 (0.263)	1.106 (0.227) *	0.225 (0.212)	0.153 (0.212)	0.008	4.33 (<0.0001)
Uninsured	1.454 (0.765)	1.705 (0.806)	2.352 (0.902)	2.904 (0.880) *	3.191 (0.758) *	3.724 (0.709) *	2.335 (0.709) *	0.011	5.74 (<0.0001)
Health Status/Outcomes									
Cancer	0.092 (0.094)	−0.047 (0.099)	0.101 (0.110)	−0.062 (0.108)	0.241 (0.093)	0.063 (0.087)	−0.038 (0.087)	0.003	2.43 (0.02)
Chronic obstructive pulmonary disease	0.179 (0.114)	0.069 (0.120)	0.256 (0.134)	0.319 (0.131)	0.770 (0.113) *	0.599 (0.106) *	0.275 (0.105)	0.018	8.89 (<0.0001)
Coronary heart disease	0.293 (0.119)	0.224 (0.126)	0.428 (0.141)	0.312 (0.137)	0.725 (0.118) *	0.669 (0.111) *	0.346 (0.111)	0.017	8.17 (<0.0001)
Diabetes	0.597 (0.256)	0.602 (0.269)	0.654 (0.031)	0.973 (0.294) *	1.463 (0.253) *	1.473 (0.237) *	0.843 (0.237) *	0.016	7.69 (<0.0001)
Obesity	0.539 (0.370)	0.580 (0.390)	0.884 (0.436)	1.268 (0.425)	3.223 (0.366) *	2.601 (0.343) *	1.415 (0.343) *	0.031	14.66 (<0.0001)
Poor mental health status	0.236 (0.174)	0.253 (0.183)	0.406 (0.205)	0.618 (0.200)	0.799 (0.172) *	0.749 (0.161) *	0.462 (0.161)	0.009	5.32 (<0.0001)
Poor physical health status	0.577 (0.257)	0.552 (0.271)	0.922 (0.303)	0.909 (0.296)	1.511 (0.255) *	1.537 (0.238) *	0.881 (0.238) *	0.018	8.54 (<0.0001)

* *p* ≤ 0.001 based on a Bonferroni correction for multiple tests. ^a^ Reference group is long-term high; ^b^ Reference group is Quartile 4 (highest); ^c^ log-transformed.

## Data Availability

The Agency for Healthcare Research and Quality Social Determinants of Health dataset from 2010 is openly available at https://www.ahrq.gov/sdoh/data-analytics/sdoh-data.html (accessed on 29 May 2025); the 2017 version of the 500 Cities Project is openly available at https://data.cdc.gov/browse?category=500+Cities+%26+Places&q=&sortBy=relevance&page=1&pageSize=20 (accessed on 29 May 2025); the Neighborhood Change Database can be purchased from Geolytics here: https://geolytics.com/neighborhood-change-database-2010 (accessed on 29 May 2025).

## Supplementary Materials

The following are available online at https://www.mdpi.com/article/10.3390/ijerph22071076/s1, Table S1. Measures, Definitions, Data Source and Years of Data collection. Table S2. Bivariate associations between social determinants of health, health-related characteristics, and trajectories, N = 2961 census tracts, Texas. Table S3. Linear regression models examining associations between Black trajectories and social determinants of health and health-related characteristics, N = 2961 census tracts, Texas. Table S4. Linear regression models examining associations between Hispanic trajectories and social determinants of health and health-related characteristics, N = 2961 census tracts, Texas. Figure S1. Distribution of longitudinal White trajectories by longitudinal poverty trajectories, census tracts, TX (1990–2010).
