# Supplementary material for: A Longitudinal Ecologic Analysis of Neighborhood-Level Social Inequalities in Health in Texas
Source: Int J Environ Res Public Health. 2025 Jul 5;22(7):1076. doi: 10.3390/ijerph22071076 (PMC12294406; doi:10.3390/ijerph22071076)

Table S1. Measures, Definitions, Data Source and Years of Data collection

Measure	Definition	Data Source	Year (s)
**Social Determinants of Health**			
Distance to the nearest emergency department	Calculated using population weighted tract centroids to determine the distance to the closest emergency department for a given area (miles).	Centers for Medicare and Medicaid Provider of Services File	2010
Distance to the nearest obstetrics department	Calculated using population weighted tract centroids to determine the distance to the closest obstetrics department for a given area (miles).	Centers for Medicare and Medicaid Provider of Services File	2010
Median home value ($)	The middle value of all the estimated market values of homes that are currently occupied by their owners within a geographic area ($).	American Community Survey	2010
Rented occupied housing units (%)	Occupied housing units that are not owner-occupied.	American Community Survey (ACS)	2010
Crowded housing (%)	Housing units with more than 1.5 occupants per room, dividing the number of people living in the housing unit by the number of rooms.	American Community Survey (ACS)	2010
Gini index of income inequality	Summary measure of income inequality across the entire income distribution. It quantifies income inequality on a scale from 0 to 1, where 0 indicates perfect equality (everyone has the same income) and 1 represents perfect inequality (one person holds all the income). This measure is based on the difference between the Lorenz curve (the observed cumulative income distribution) and the notion of a perfectly equal income distribution.	American Community Survey	2010

Less than high school	People aged 25 and older who not have a high school diploma.	American Community Survey (ACS)	2010
Bachelor's degree or higher	People aged 25 and older with a bachelor’s degree or more education.	American Community Survey (ACS)	2010
Public assistance income or food stamps/SNAP	Public assistance income includes cash and non-cash benefits such as food stamps (SNAP), Temporary Assistance for Needy Families (TANF) and Supplemental Security Income (SSI).	American Community Survey (ACS)	2010
Unemployed (%)	Those who do not have a job and have actively looked for work in the past four weeks.	American Community Survey (ACS)	
Limited English proficiency	No member of the household 14 years and over who speaks only English or speaks English “very well”.	American Community Survey	2010
Single-parent households	A household where only one parent is present and there are one or more children under the age of 18 living in the household.	American Community Survey (ACS)	2010
**Health Risk & Preventive Behaviors (%)**			
Insufficient sleep	Less than seven hours of sleep per night on average.	500 cities, 2017/BRFSS	2014
Current smoking	Smoked at least 100 cigarettes in one's lifetime and currently smoking every day or some days.	500 cities, 2017/BRFSS	2015
No physical Activity	Not participating in any leisure-time physical activities outside of work in the past month, such as running, walking for exercise or gardening.	500 cities, 2017/BRFSS	2015
Hypertension	Ever been told by a doctor or other healthcare professional that they have	500 cities, 2017/BRFSS	2015

	high blood pressure (blood pressure reading of 130/80 mm Hg or higher).		
Dental Visit	Prevalence of adults visiting a dentist or dental clinic within the past year.	500 cities, 2017/BRFSS	2014
Routine checkup	The percentage of adults having received a general physical exam from a doctor within the previous year.	500 cities, 2017/BRFSS	2015
Uninsured	An individual who lacks coverage from private health insurance, Medicare, Medicaid, CHIP, military plans, or other government programs, or if they only have Indian Health Service (IHS) coverage.	500 cities, 2017/BRFSS	2015
**Health Status/Outcomes (%)**			
Cancer	Adults diagnosed with cancer, excluding non-melanoma skin cancer, by a healthcare professional.	500 cities, 2017/BRFSS	2015
Chronic obstructive pulmonary disease	Adults diagnosed with COPD, emphysema, or chronic bronchitis by a healthcare professional.	500 cities, 2017/BRFSS	2015
Coronary Heart disease	Adults diagnosed with angina or coronary heart disease by a doctor, nurse, or other health professional.	500 cities, 2017/BRFSS	2015
Diabetes	Adults who have been told by a doctor or other health professional that they have diabetes, excluding cases of diabetes diagnosed only during pregnancy for female respondents.	500 cities, 2017/BRFSS	2015
Obesity	Adults who have a body mass index (BMI) of 30.0 kg/m^2 or higher, based on self-reported height and weight.	500 cities, 2017/BRFSS	2015

Poor mental health status	Adults who report experiencing poor mental health—including stress, depression, and emotional problems—for 14 or more days within the past 30 days.	500 cities, 2017/BRFSS	2015
Poor physical health status	Adults who report experiencing poor physical health—including illness or injury—for 14 or more days within the past 30 days.	500 cities, 2017/BRFSS	2015
**Poverty Trajectories (%)**			
Long-term low	All time periods were either low (<5%) or a combination of low and moderate (5-15%) poverty with no discernible pattern.	Neighborhood Change Database (NCB)	1990-2010
Long-term moderate	All time periods were moderate (5-15%) poverty.	Neighborhood Change Database (NCB)	1990-2010
Long-term high	All time periods were either high (>20%) or a combination of high and moderate poverty with no discernible pattern.	Neighborhood Change Database (NCB)	1990-2010
Increasing	Low or moderate poverty in 1990, increased to moderate or high after 1990.	Neighborhood Change Database (NCB)	1990-2010
Decreasing	Moderate or high poverty in 1990, decreased to low or moderate after 1990.	Neighborhood Change Database (NCB)	1990-2010
**White trajectories (%)**			
Long-term low	Proportions of non-Hispanic White concentration where all three time periods were either low (lowest tertile) or a combination of low and moderate (middle tertile) with no discernable pattern.	Neighborhood Change Database (NCB)	1990-2010
Long-term moderate	Proportions of non-Hispanic White concentration where all three time periods were moderate.	Neighborhood Change Database (NCB)	1990-2010

Long-term high	Proportions of non-Hispanic White concentration where all three time periods were either high (highest tertile) or a combination of high and moderate with no discernable pattern.	Neighborhood Change Database (NCB)	1990-2010
Increasing	Proportions of non-Hispanic White concentration that was low or moderate in 1990, increased to moderate or high after 1990.	Neighborhood Change Database (NCB)	1990-2010
Decreasing	Proportions of non-Hispanic White concentration that was moderate or high in 1990, decreased to low or moderate after 1990.	Neighborhood Change Database (NCB)	1990-2010

Table S2. Bivariate associations between social determinants of health, health-related characteristics, and trajectories, N=2,961 census tracts, Texas

**Social Determinants of Health**	**Poverty Trajectories** (anova or chi-square test significant?)	**White Trajectories** (anova or chi-square test significant?)	**Black Trajectories** (anova or chi-square test significant?)	**Hispanic Trajectories** (anova or chi-square test significant?)
Distance to the nearest emergency department	Yes	Yes	Yes	Yes
Distance to the nearest obstetrics department	Yes	Yes	Yes	Yes
Median home value	Yes	Yes	Yes	Yes
Rented occupied housing units	Yes	Yes	Yes	Yes
Crowded housing	Yes	Yes	Yes	Yes
Gini index of income inequality	Yes	Yes	Yes	Yes
Less than high school	Yes	Yes	Yes	Yes
Bachelor's degree	Yes	Yes	Yes	Yes
Public assistance income or food stamps/SNAP	Yes	Yes	Yes	Yes
Unemployed	Yes	Yes	Yes	Yes
Limited English proficiency	Yes	Yes	Yes	Yes
Single-parent households	Yes	Yes	Yes	Yes
**Health Risk & Preventive Behaviors**				
Insufficient sleep	Yes	No	No	Yes

Current smoking	No	Yes	Yes	Yes
No physical activity	Yes	No	Yes	No
Hypertension	Yes	No	Yes	Yes
Dental Visit	Yes	No	Yes	Yes
Routine checkup	Yes	No	No	Yes
Uninsured	Yes	No	Yes	No
**Health Status/Outcomes**				
Cancer	Yes	No	No	No
Chronic obstructive pulmonary disease	Yes	No	Yes	Yes
Coronary Heart disease	Yes	No	Yes	No
Diabetes	Yes	No	Yes	No
Obesity	Yes	Yes	No	No
Poor mental health status	Yes	No	Yes	No
Poor physical health status	Yes	No	Yes	No

Table S3. Linear regression models examining associations between Black trajectories and social determinants of health and health-related characteristics, N=2,961 census tracts, Texas

	Long-term Moderate[a]	Long-term High[a]	Increasing[a]	Decreasing[a]	Population Density (quartile 1)[b]	Population Density (quartile 2)[b]	Population Density (quartile 3)[b]	R^2	F (p value)
	beta (se)	beta (se)	beta (se)	beta (se)	beta (se)	beta (se)	beta (se)		
**Social Determinants of Health**									
Distance to the nearest emergency department[c]	-0.144 (0.049)	0.008 (0.045)	-0.215 (0.053)*	-0.126 (0.053)	1.087 (0.045)*	0.430 (0.044)*	0.114 (0.044)	0.210	110.31 (<.0001)
Distance to the nearest obstetrics department[c]	-0.124 (0.046)	-0.085 (0.042)	-0.079 (0.049)	-0.089 (0.050)	1.080 (0.042)*	0.230 (0.041)*	0.055 (0.042)	0.239	129.83 (<.0001)
Median home value[c]	0.085 (0.032)	-0.252 (0.030)*	0.264 (0.034)*	-0.094 (0.035)	-0.165 (0.029)*	0.056 (0.029)	0.038 (0.029)	0.112	52.80 (<.0001)
Rented occupied housing units	4.243 (1.196)*	16.070 (1.105)*	7.113 (1.291)*	5.957 (1.297)*	-23.998 (1.103)*	-16.222 (1.084)*	-12.774 (1.085)*	0.224	119.15 (<.0001)
Crowded housing[c]	-0.271 (0.126)	0.834 (0.116)*	-0.565 (0.136)*	0.566 (0.136)*	-0.525 (0.116)*	-0.961 (0.114)*	-0.755 (0.114)*	0.083	38.25 (<.0001)
Gini index of income inequality	-0.026 (0.004)*	-0.015 (0.004)*	-0.046 (0.004)*	-0.014 (0.004)	-0.001 (0.004)	-0.008 (0.004)	-0.006 (0.004)	0.045	20.33 (<.0001)
Less than high school	-8.423 (0.887)*	-0.261 (0.820)	13.560 (0.957)*	1.952 (0.962)	-7.487 (0.818)*	-6.871 (0.804)*	-5.659 (0.805)*	0.146	71.14 (<.0001)
Bachelor's degree	3.348 (0.655)*	-5.228 (0.605)*	7.957 (0.707)*	-2.560 (0.711)*	-2.890 (0.604)*	2.069 (0.594)*	2.010 (0.595)*	0.167	83.69 (<.0001)
Public assistance income or food stamps/SNAP[c]	-0.319 (0.094)*	0.780 (0.086)*	-0.764 (0.101)*	0.175 (0.102)	-0.046 (0.086)	-0.480 (0.085)*	-0.359 (0.085)*	0.113	53.27 (<.0001)
Unemployed	-0.852 (0.240)*	2.700 (0.221)*	-1.132 (0.259)*	0.095 (0.260)	-1.156 (0.221)*	0.385 (0.217)	0.037 (0.218)	0.127	60.62 (<.0001)
Limited English proficiency[c]	-0.285 (0.111)	0.078 (0.103)	-0.414 (0.120)*	0.312 (0.121)	-1.957 (0.102)*	-1.146 (0.101)*	-0.727 (0.101)*	0.129	61.85 (<.0001)

Single-parent households	0.143 (0.968)	14.538 (0.895)*	-1.710 (1.045)	2.490 (1.050)	-8.596 (0.893)*	-4.697 (0.878)*	-3.567 (0.879)*	0.168	83.57 (<.0001)
**Health Risk & Preventive Behaviors**									
Insufficient sleep	-0.121 (0.267)	0.417 (0.244	-0.007 (0.286)	-0.213 (0.286)	1.978 (0.242)*	1.483 (0.239)*	0.878 (0.240)*	0.025	11.77 (<.0001)
Current smoking	0.143 (0.289)	0.801 (0.265)	0.258 (0.310)	-0.445 (0.310)	1.664 (0.262)*	1.083 (0.259)*	0.374 (0.260)	0.017	8.29 (<.0001)
No physical activity	0.483 (0.489)	1.742 (0.448)*	1.322 (0.525)	0.804 (0.524)	3.192 (0.443)*	2.973 (0.439)*	1.716 (0.440)*	0.024	11.48 (<.0001)
Hypertension	0.222 (0.432)	1.206 (0.446)	0.051 (0.459)	0.746 (0.507)	2.943 (0.372)*	2.290 (0.362)*	1.013 (0.362)	0.026	12.14 (<.0001)
Dental Visit	-0.955 (0.800)	-2.855 (0.734)*	-0.814 (0.860)	-0.734 (0.859)	-3.351 (0.727)*	-3.206 (0.719)*	-1.629 (0.722)	0.012	6.24 (<.0001)
Routine checkup	-0.195 (0.234)	-0.267 (0.214)	-0.313 (0.251)	0.176 (0.251)	0.912 (0.212)*	0.211 (0.210)	0.145 (0.211)	0.008	4.43 (<.0001)
Uninsured	1.336 (0.781)	3.052 (0.716)*	0.950 (0.839)	1.678 (0.838)	3.022 (0.709)*	3.544 (0.701)*	2.218 (0.704)	0.013	6.71 (<.0001)
**Health Status/Outcomes**									
Cancer	-0.034 (0.096)	0.038 (0.088)	-0.088 (0.103)	0.105 (0.103)	0.229 (0.087)	0.062 (0.086)	-0.040 (0.086)	0.003	2.25 (<0.001)
Chronic obstructive pulmonary disease	0.106 (0.116)	0.413 (0.106)*	0.133 (0.125)	0.168 (0.125)	0.746 (0.105)*	0.561 (0.104)*	0.253 (0.105)	0.021	10.23 (<.0001)
Coronary Heart disease	0.155 (0.122)	0.527 (0.111)*	0.076 (0.131)	0.380 (0.130)	0.704 (0.110)*	0.648 (0.109)*	0.335 (0.110)	0.023	10.89 (<.0001)
Diabetes	0.411 (0.261)	1.169 (0.239)*	0.259 (0.280)	0.829 (0.280)	1.373 (0.236)*	1.422 (0.234)*	0.820 (0.235)*	0.021	10.22 (<.0001)
Obesity	0.390 (0.378)	1.071 (0.346)	0.308 (0.406)	0.608 (0.405)	3.118 (0.343)*	2.526 (0.339)*	1.367 (0.341)*	0.032	14.77 (<.0001)
Poor mental health status	0.254 (0.177)	0.615 (0.163)*	0.262 (0.191)	0.169 (0.190)	0.768 (0.161)*	0.691 (0.159)*	0.426 (0.160)	0.012	5.91 (<.0001)
Poor physical health status	0.478 (0.262)	1.179 (0.240)*	0.325 (0.282)	0.669 (0.281)	1.466 (0.238)*	1.469 (0.235)*	0.837 (0.236)*	0.022	10.53 (<.0001)

*p<=0.001 based on a Bonferroni correction for multiple tests

[a] Reference group is long-term low; [b] Reference group is Quartile 4 (highest); [c] log-transformed

Table S4. Linear regression models examining associations between Hispanic trajectories and social determinants of health and health-related characteristics, N=2,961 census tracts, Texas

	Long-term Moderate[a]	Long-term High[a]	Increasing[a]	Decreasing[a]	Population Density (quartile 1)[b]	Population Density (quartile 2)[b]	Population Density (quartile 3)[b]	R^2	F (p value)
	beta (se)	beta (se)	beta (se)	beta (se)	beta (se)	beta (se)	beta (se)		
**Social Determinants of Health**									
Distance to the nearest emergency department[c]	0.341 (0.048)*	0.241 (0.045)*	0.218 (0.054)*	0.107 (0.056)	1.143 (0.046)*	0.453 (0.045)*	0.117 (0.045)	0.218	115.31 (<.0001)
Distance to the nearest obstetrics department[c]	-0.009 (0.045)	-0.033 (0.042)	-0.046 (0.051)	-0.108 (0.053)	1.089 (0.043)*	0.225 (0.042)*	0.044 (0.042)	0.238	129.26 (<.0001)
Median home value[c]	-0.347 (0.028)*	-0.742 (0.027)*	-0.536 (0.032)*	0.022 (0.033)	-0.354 (0.027)*	-0.063 (0.026)	-0.023 (0.026)	0.286	165.35 (<.0001)
Rented occupied housing units	7.501 (1.199)*	9.778 (1.135)*	8.762 (1.371)*	8.234 (1.408)*	-23.995 (1.157)*	-14.492 (1.127)*	-11.757 (1.121)*	0.183	92.94 (<.0001)
Crowded housing[c]	1.596 (0.111)*	2.759 (0.105)*	2.282 (0.127)*	0.468 (0.131)*	0.155 (0.107)	-0.528 (0.105)*	-0.505 (0.104)*	0.249	136.75 (<.0001)
Gini index of income inequality	-0.008 (0.004)	0.003 (0.004)	-0.013 (0.005)	0.015 (0.005)	0.002 (0.004)	-0.010 (0.004)	-0.008 (0.004)	0.017	8.14 (<.0001)
Less than high school	7.201 (0.697)*	26.763 (0.659)*	15.415 (0.797)*	1.187 (0.818)	0.688 (0.672)	-2.632 (0.655)*	-3.227 (0.651)*	0.449	335.01 (<.0001)
Bachelor's degree	-9.401 (0.549)*	-18.320 (0.520)*	-13.884 (0.628)*	0.0416 (0.645)	-7.705 (0.530)*	-0.669 (0.516)	0.659 (0.514)	0.388	260.68 (<.0001)
Public assistance income or food stamps/SNAP[c]	1.126 (0.085)*	2.154 (0.080)*	1.608 (0.097)*	0.355 (0.099)*	0.472 (0.082)*	-0.140 (0.079)	-0.181 (0.079)	0.243	132.44 (<.0001)
Unemployed	0.799 (0.242)*	2.123 (0.229)*	1.761 (0.277)*	-0.964 (0.284)*	-0.753 (0.233)	-0.001 (0.227)	0.016 (0.226)	0.070	31.83 (<.0001)
Limited English proficiency[c]	1.508 (0.093)*	2.782 (0.088)*	2.102 (0.106)*	0.816 (0.109)*	-1.207 (0.090)*	-0.686 (0.087)*	-0.413 (0.087)*	0.362	233.49 (<.0001)

Single-parent households	7.331 (0.968)*	10.111 (0.895)*	8.744 (1.045)*	1.736 (1.050)	-7.673 (0.893)*	-3.162 (0.878)*	-3.176 (0.879)*	0.086	39.41 (<.0001)
**Health Risk & Preventive Behaviors**									
Insufficient sleep	0.482 (0.259)	0.284 (0.245)	0.480 (0.286)	-0.475 (0.296)	2.045 (0.248)*	1.563 (0.242)*	0.880 (0.242)*	0.027	12.60 (<.0001)
Current smoking	0.013 (0.281)	-0.448 (0.266)	0.458 (0.320)	-0.360 (0.324)	1.522 (0.269)*	1.075 (0.263)*	0.322 (0.262)	0.016	7.98 (<.0001)
No physical activity	0.560 (0.476)	0.511 (0.451)	1.144 (0.543)	0.315 (0.549)	3.156 (0.456)*	3.068 (0.446)*	1.733 (0.445)*	0.020	9.49 (<.0001)
Hypertension	0.252 (0.391)	0.253 (0.370)	1.091 (0.446)	-1.077 (0.451)	3.028 (0.374)*	2.163 (0.366)*	0.981 (0.365)	0.027	12.78 (<.0001)
Dental Visit	-0.613 (0.779)	0.490 (0.738)	-1.730 (0.890)	-0.754 (0.900)	-2.943 (0.746)*	-3.197 (0.729)*	-1.555 (0.728)	0.009	4.91 (<.0001)
Routine checkup	0.224 (0.226)	0.397 (0.214)	0.237 (0.258)	-0.969 (0.262)*	1.123 (0.217)*	0.255 (0.212)	0.142 (0.211)	0.017	8.39 (<.0001)
Uninsured	0.933 (0.761)	0.781 (0.720)	1.531 (0.868)	1.977 (0.878)	2.765 (0.729)*	3.623 (0.712)*	2.277 (0.711)	0.009	4.76 (<.0001)
**Health Status/Outcomes**									
Cancer	0.067 (0.093)	0.130 (0.088)	0.193 (0.106)	-0.161 (0.107)	0.286 (0.089)	0.081 (0.087)	-0.037 (0.087)	0.006	3.37 (<0.001)
Chronic obstructive pulmonary disease	0.112 (0.113)	0.038 (0.107)	0.332 (0.129)	-0.312 (0.130)	0.737 (0.108)*	0.583 (0.106)*	0.250 (0.106)	0.020	9.44 (<.0001)
Coronary Heart disease	0.195 (0.118)	0.182 (0.112)	0.438 (0.135)	-0.0317 (0.137)	0.716 (0.113)*	0.671 (0.111)*	0.333 (0.111)	0.018	8.59 (<.0001)
Diabetes	0.399 (0.254)	0.288 (0.241)	0.775 (0.290)	-0.090 (0.293)	1.348 (0.243)*	1.451 (0.238)*	0.806 (0.237)*	0.015	7.44 (<.0001)
Obesity	0.612 (0.368)	0.220 (0.348)	0.554 (0.420)	-0.179 (0.424)	3.047 (0.352)*	2.532 (0.334)*	1.334 (0.343)*	0.030	13.98 (<.0001)
Poor mental health status	0.188 (0.173)	0.019 (0.163)	0.343 (0.197)	0.194 (0.199)	0.697 (0.165)*	0.710 (0.162)*	0.430 (0.161)	0.008	4.33 (<.0001)
Poor physical health status	0.398 (0.256)	0.300 (0.242)	0.690 (0.292)	0.239 (0.295)	1.409 (0.245)*	1.509 (0.235)*	0.845 (0.236)	0.015	7.51 (<.0001)

*p<=0.001 based on a Bonferroni correction for multiple tests

[a] Reference group is long-term low; [b] Reference group is Quartile 4 (highest); [c] log-transformed

Figure S1. Distribution of longitudinal White trajectories by longitudinal poverty trajectories, census tracts, TX (1990-2010)

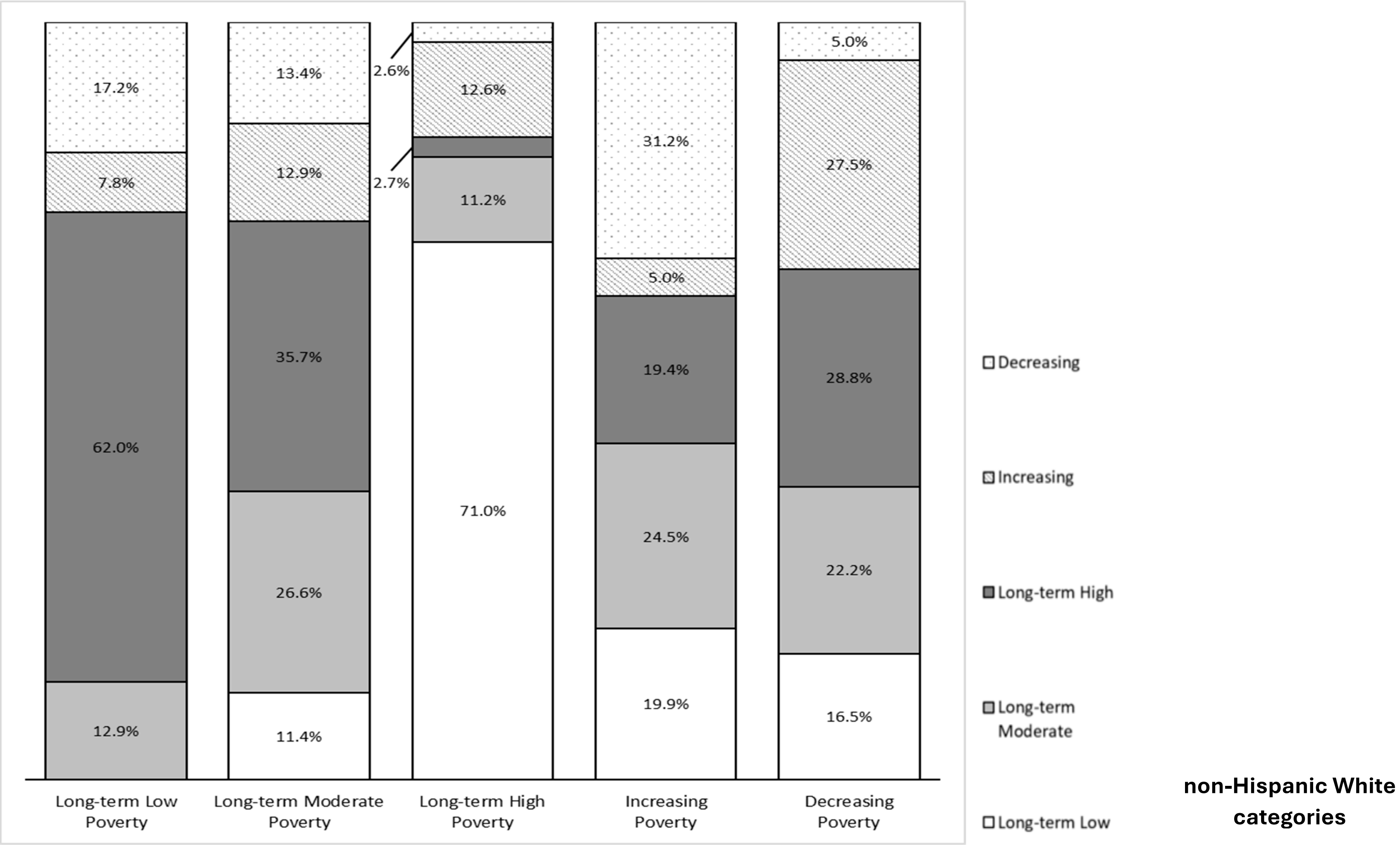

Supplement: Supplementary file 1 [file ijerph-22-01076-s001.zip › ijerph-3703491-supplementary.pdf]
